# Supplementary material for: Cannabidiol interactions with voltage-gated sodium channels
Source: eLife. 2020 Oct 22;9:e58593. doi: 10.7554/eLife.58593 (PMC7641581; doi:10.7554/eLife.58593)
Supplement: Supplementary file 1. [file elife-58593-supp1.docx]

**Supplementary File 1: Crystal Structure Parameters.**

| *Data collection* | **NavMs_L_ (PDB ID 6YZ2)** | **NavMs_L_-CBD (PDB ID 6YZ0)** |
| --- | --- | --- |
| Wavelength (Å) | 0.97624 | 0.97620 |
| Space group | I422 | I422 |
| *Unit-cell parameters* |  |  |
| a, b, c (Å) | 108.94 ,108.94,209.37 | 110.4,110.4,207.8 |
| α, β, γ (^o^) | 90, 90, 90 | 90, 90, 90 |
| Resolution range (Å) | 104.68-2.20 (2.27-2.20) | 103.94-2.30 (2.38-2.30) |
| Total number of observations | 687781 (56721) | 603236 (58839) |
| Total number unique | 32388 (2771) | 28708 (2770) |
| Completeness | 100.0 (100.0) | 99.3 (100.0) |
| Multiplicity | 21.2 (20.5) | 21.0 (21.2) |
| <*I/σ(I)>* | 17.4 (2.2) | 17.3 (1.0) |
| CC(1/2) | 0.999 (0.950) | 0.999 (0.877) |
| R_merge all_ | 0.093 (1.20) | 0.085 (3.803) |
| R_pim All_ | 0.021 (0.278) | 0.020 (0.877) |
| Solvent content (%) | 72.80 | 73.64 |
| Molecule per ASU | 1 | 1 |
| Wilson B factor (Å^2^) | 46.9 | 52.6 |
| *Refinement* |  |  |
| Resolution Range (Å) | 28.8-2.2 | 48.1-2.3 |
| R_work_ | 0.24 | 0.23 |
| R_free_ | 0.26 | 0.26 |
| Reflection, working | 32388 | 27254 |
| Reflection, free | 1619 | 1376 |
| Average B factor (all atoms) | 75.0 | 95.3 |
| RMS bond angle | 0.82 | 0.90 |
| RMS bond length (Å) | 0.008 | 0.009 |
| *Ramachandran Analysis:* |  |  |
| Preferred region (%) | 96.6 | 95.8 |
| Allowed region (%) | 3.0 | 4.2 |
| Outliers (%) | 0.4 | 0.0 |
